# When and Why Contexts Predict Unethical Behavior: Evidence From a Laboratory Bribery Game

**DOI:** 10.3389/fpsyg.2021.675319

**Published:** 2021-07-09

**Authors:** Sining Wang, Tao Chen

**Affiliations:** ^1^Department of Economics, Case Western Reserve University, Cleveland, OH, United States; ^2^Big Data Research Lab, Department of Economics, University of Waterloo, Waterloo, ON, Canada

**Keywords:** unethical decision, context effect, bribery game, corruption, experimental design

## Abstract

In economic unethical decision-making experiments, one important methodological investigation is what types of contexts should be used to frame the instructions. Within the experimental economics community, using neutral-context instructions instead of loaded-context instructions is the mainstream practice. Because the loaded contexts may impact behavior in an unpredictable manner and therefore, put experimental control at risk. Nevertheless, using the loaded-context instructions could be advantageous in several ways. A properly framed context can help to facilitate learning and gain ecological validity. The challenge is whether we can identify when and why the loaded context may alter behavior. In this paper, we aim to test if being familiar with a loaded context can systematically influence unethical decisions in a bribery game. We conduct a laboratory bribery game experiment with three different treatments: the neutral-context treatment, the familiar-context treatment, and the unfamiliar-context treatment. Using the neutral-context treatment as a benchmark, we find that participants in the familiar-context treatment express stronger negative attitudes toward corruption. Attitudes toward unethical behavior are the same in the neutral-context treatment and the unfamiliar-context treatment. Behaviorally, the participants in the familiar-context treatment are much less likely to engage in corrupt activities. The neutral-context treatment and the unfamiliar-context treatment produce the same behavioral outcome.

## Introduction

Over the past three decades, the study of unethical decision making has received increasing attention. In laboratory economic experiments, one commonly used technique to investigate the underlying motivation of unethical behavior is to put a decision maker in a position where he or she must decide whether to engage in economically rational but dishonest practices. In such experiments, an important methodological debate is whether one should frame the experimental instruction with neutral context or loaded context (Alekseev et al., 2017).

Within the experimental economics community, framing the instruction with neutral context is the mainstream practice. Smith (1976) proposed that people with varied backgrounds and preferences may interpret the value of ethics embedded in the context differently. The different interpretations are often unobservable, and therefore, will affect behavior in an unpredictable manner. To avoid uncontrollable data distortion, experimenters should use “neutralized” instruction, and then induce the subjects’ preferences with only monetary reward. However, this approach has been criticized because it focuses solely on the external incentives thus ignoring the importance of ethics and psychological costs (Bardhan, 2006). A large literature also suggests that using loaded context instruction could be advantageous—a meaningful context that is related with the research question can help the researcher better understand the participants’ motives (Alm et al., 1992; Aronson, 1992, 1999; Andreoni, 1995; Andreoni and Miller, 2002; Abbink and Hennig-schmidt, 2006; Bardhan, 2006; Alatas et al., 2009; Barr and Serra, 2009; Armantier and Boly, 2014; Banerjee, 2016; Alekseev et al., 2017). Moreover, the loaded context can facilitate learning, making the experimental tasks more understandable to the participants (Wason and Shapiro, 1971; Griggs and Cox, 1982; Chou et al., 2009).

It is generally agreed that altering the experimental context could have profound effects on unethical decisions. The bone of contention is whether such effects are predictable. Many past studies have contributed to this heated and ongoing debate, yet little consensus has been reached. For instance, it is presumably that context plays a major role in determining people’s decisions in bribery games—calling participants “Public officers” and “Firm owners” instead of “Player 1” and “Player 2” may lead to divergent behavioral outcomes. As a matter of fact, a considerable amount of evidence has been found to support this conjecture (Eckel and Grossman, 1996; Cooper et al., 1999; Carpenter et al., 2008; Laury and Taylor, 2008; Alatas et al., 2009; Barr and Serra, 2009). However, multiple studies show that the neutral context and the loaded context produce the same behaviors in bribery games (Cooper et al., 1999; Barr and Serneels, 2004; Abbink and Hennig-schmidt, 2006; Armantier and Boly, 2014).

The question we address in this paper is whether the effect of context is always unpredictable. In particular, we use a laboratory bribery game as an example to examine what kind of experimental context may influence unethical decisions in a systematic, predictable way.

## Not All Contexts are Created Equal

Past studies in bribery games examine the distinctions between two types of contexts: either neutral context (framed with abstract language, no specific background story) or loaded context (framed with a specific background story). However, we consider such a dichotomous view insufficient: Not all the loaded contexts have the same impact on decisions. Extensive evidence suggests that emotional responses triggered by the context alter people’s behavior. It is worthwhile to take account of how people’s real-life experiences may influence their perceptions of the loaded contexts, which in turn, affect decision.

Alekseev et al. (2017) proposed to distinguish between three types of contexts. The first type, which is called the “abstract context” or “neutral context,” uses neutral language such as “player A,” “option B” and so on: The neutral context is not related to any specific background story. The second type, which is called the “meaningful context,” presents the experimental tasks in specific scenarios. However, the artificial scenarios do not evoke emotions or connotations. The third type, which is called the “evocative context,” presents the tasks in scenarios that are not only related to a real-life situation, but also evoke strong emotional responses. Inspired by this insight, we consider people’s emotional responses might be the key to understand the mechanism through which contexts affect people’s decision in unethical decision-making experiments.

From the psychology literature, Blanchette and Caparos (2013) showed that emotion plays a significant role in logical reasoning and decision making. In particular, they suggested that contexts that is relevant to individual’s past experiences are more likely to evoke emotional responses. Consequently, people tend to devote more cognitive resources to such decision-making situations. To put it in another way, a decision-maker would be more “emotional” in contexts that is relevant to themselves. Goel and Vartanian (2011) compared people’s reasoning process in neutral contexts and emotionally charge contexts. They found that under certain conditions, the emotional factors in the context can foster a more vigilant, systematic information-processing style. Greene et al. (2001) investigated the changes in brain activities when people respond to ethical dilemmas. The same ethical dilemma was presented in two contexts: personal context (where the participants are more engaged emotionally) and impersonal context (where the participants are less engaged emotionally). They found that responding to personal ethical dilemmas produces increased brain activity in areas associated with emotional processing. Besides, they also found people have to spend more cognitive resources to overcome their emotional responses in the personal context.

All the above studies lead to the point that emotion and context jointly determine behavior. In the realm of unethical decision-making, we argue that an evocative context may alter people’s reasoning and behavior by increasing the emotional charge. For instance, in bribery games, unethical behaviors typically impose negative externalities to the society, which might bring the individual with considerable psychological costs. Adopting the evocative context may make the psychological costs more salient. When people are facing scenarios that evoke strong (negative) emotional responses, they are more likely to think about the negative consequences of their decision. Accordingly, their behaviors in the lab can better reflect what they may do in naturally occurring environments in their everyday life.

In the current study, we aim to test if being familiar with a loaded context can systematically influence unethical decisions in a bribery game. In particular, we put forward that a loaded context that is closely related to the decision maker’s real-life experience is more likely to orient her to associate the hypothetical scenario with her self-concept, and therefore, evoke strong emotional responses. Consequently, the decision maker is more likely to perceive it as the “evocative context” (and putatively more emotional). The decision maker is more engaged with the task and is likely to devote more attention to her decisions. Moreover, the moral standard and social norms embedded in such a context are more salient to the individual. Actions that violate certain moral obligations or injunctive norms would bring the decision maker with considerable psychological costs. Behaviorally, the decision maker is less likely to engage in dishonest practices.

**Hypothesis 1.** Unethical behaviors should be less likely to happen in the evocative context, as compared with the meaningful context and the neutral context.

On the other hand, a loaded context that is distant from the individual’s real-life experience is more likely to be perceived as the “meaningful context” (and putatively less emotional). Although the meaningful context is constructed with a specific scenario, it doesn’t evoke strong emotions or significant psychological responses—It leads the decision maker to be unattached to the task. The moral standards and social norms in a meaningful (yet remote) context are ambiguous, or vague to the individual. The ambiguity in the moral standard plus the lack of personal involvement make it easier to find external justifications for a dishonest practice. To escape from the aversive state and strive for self-consistency, people would rationalize their unethical decisions. The reasoning can possibly be: “This is just a game; I would not do that in real life” (although the participant had no similar experience in real life), or “I’m curious about what the consequences are for choosing this; let me try it out.” Because neither the meaningful context nor the neutral context evokes strong emotions, the psychological cost of engaging in dishonest behavior should be similar in these two conditions. Thus, we would expect the meaningful context and the neural context drives similar behavioral outcome.

**Hypothesis 2.** The meaningful context and the neutral context will lead to similar behavioral outcomes.

While people’s behaviors are observable, the motives of the behaviors are not. In the field of social psychology, it has been widely accepted that behavior is guided by attitudes (e.g., Ajzen et al., 2018). In the current study, we are curious about if attitudes toward bribery can help explain unethical decision making. To complement the laboratory experiment, we conduct an independent survey to measure participants’ attitude toward bribery. We want to test if people’s attitude toward corruption can predict behavior in the bribery game.

## Experimental Design

### Administration

The experiment is conducted at the school of business in Jianghan University (Wuhan, China). The experimental procedure is reviewed and approved by the research ethics committee at Jianghan University. To recruit participants, we distribute recruitment flyer to students during their self-study sessions. Students who are interested in participating will response to our recruit email on the flyer. The experimenter then sends them the electronic copy of the information sheet. Potential participants can take as much time as they need to make the decision. For students who decided to participate, we send them the invitation with detailed time and location.

Upon arrival, the students will first receive the consent form, and then orally indicate whether they agree to participant. Informed consent is obtained from all participants. Next, the participant will be randomly assigned with a unique experimental identification number. This number will be used to track their decisions and responses during the experiment. Since all data are collected anonymously, we do not ask the participants to provide signed consent.

In total, 340 students (92 male, 248 female), which consisted of freshmen or sophomores, participated in the experiment. Among the 340 participants, 250 of them (56 male, 194 female) are randomly invited to our lab to play a bribery game and followed by a short questionnaire asking about their decisions and reasoning in the game. For the rest of the 90 students (36 male, 54 female), we conduct an independent attitude survey to obtain the perceived attitudes toward unethical behaviors in each game context. All data is collected anonymously. It is very important to note that each participant only participates in either the bribery game plus the corresponding questionnaire, or the attitude survey.

To run the bribery game, we conduct 13 sessions with either 10 or 20 participants in each. It takes approximately 60 min (including check-in and payment processing) to run one session. All the sessions are conducted with computer-based materials, which are developed using z-tree (Fischbacher, 2007). During the experiment, all participants make decisions anonymously, and earn “points” (the fictitious experimental currency). At the conclusion, participants are paid in cash privately at the rate: 1 RMB (=0.16 US dollar) for every 100 points they earn. The average earnings are 30 RMB (including 5 RMB show-up fee)^1^.

### The Laboratory Bribery Game

We use a laboratory bribery game to simulate a decision-making scenario in which unethical behavior may occur. All the 250 participants who participate in the bribery game are randomized into 25 groups with ten participants in each.

**FIGURE 1:**
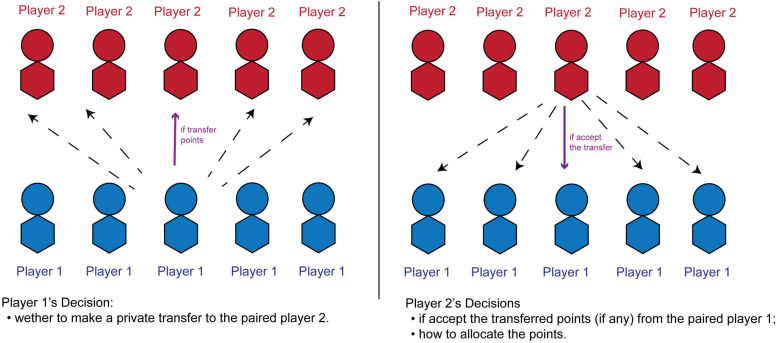
The basic setup of the players’ decisions.

In the beginning of the game, each participant is randomly assigned with a role. Within a group, five participants play as applicants (potential bribers, **player 1** below), the other five participants play as granter (potential bribee, **player 2** below). Each player 1 applies for five different grants (each grant values 1,000 game points); each player 2 is in charge of allocating the 1,000 game points among the five player 1s. In addition, each player 1 is randomly paired with a player 2. Prior to the player 2’s point allocation decision, the two participants in a pair can interact with each other. We adopt a fixed-partner design to allow repeated interactions between the paired players. All the interactions are anonymous. After the role assignment, the participants start to make decisions. The process is as follow:

• At the beginning of each period, each player 1 receives 200 points as an initial endowment. Player 2 has no endowment.
• Player 1 first decides whether to make a private ***transfer*** to the player 2 in his/her pair. If the decision is to transfer, the participant must specify a whole integer in the range from 1 to 200 points.
• Following that decision, the player 2 may face one of the two cases:
∘ Case 1: the paired player 1 decided NOT to transfer point. In this case, the player 2 sees a feedback “*no point being transferred*” and has no decision to make at this step.
∘ Case 2: the paired player 1 decided to make a transfer. Then the player 2 sees the total points being transferred by the player 1, and then decides whether to ***accept*** or ***reject*** the bribe. If accepting it, then the amount offered is deducted from the player 1’s account and added to the player 2’s account. If the player 2 rejects the bribe, then both players’ accounts remain unchanged.
• Last, the player 2 decides how to allocate the 1,000 points among the five player 1s. If abiding by the game rules, then each player 1 earns 200 points (equal split). If violating the game rules, then the player 1 in the pair earns 1,000 points, and the other player 1s earn nothing.
• After all the allocation decisions have been made, the player 1 sees feedback on how the points are allocated.
• Figure 1 illustrates the players’ decisions.

The game repeats for 15 periods with fixed partners. At the end of period 15, all participants will be reassigned with a different role, and then paired with a strange partner. The new pairs will then play the same game for another 15 periods. That is to say, if a player was the briber in the first half (period 1–15), she/he will be playing the bribee in the second half of the game (period 16–30). At the conclusion of the experiment, four periods (two from period 1–15, two from period 16–30) are randomly selected to determine the players’ payment.

**FIGURE 2:**
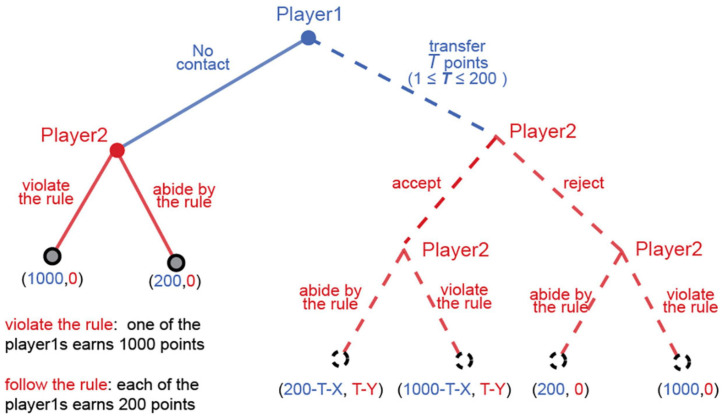
The extensive form of the bribery game in each period.

During the iterations, a pair of participants is identified as a “rule-breaking pair” if any offer from the player 1 is accepted by the player 2. If a pair has been identified as the “rule-breaking pair” at least once, then there is a 1% chance the punishment occurs: both players’ earnings are cleared from their accounts. By the end of all the 30 periods, a lottery is played out to decide whether to punish the rule-breaking pairs. The extremely low probability reflects that most corrupt activities in reality are difficult to discover. As a matter of fact, many corrupt activities are even unobservable, and the severe penalty we impose represents the consequences arising from discovery of corrupt activities. Figure 2 depicts the extensive form of the game in each period within each pair. Use *T* denotes the number of points offered by player 1. *X* and *Y* denote the possible penalty for the player 1 and the player 2, respectively.

Under the homo-economicus assumption, a rational decision maker is motivated by pure self-interest. The rational decision maker does not have to overcome moral qualms about unethical behavior. The theoretical equilibria of the game are not hard to obtain. Since this is a finite-repeated game, rational players will apply backward induction to solve for a unique subgame perfect Nash equilibrium. On an equilibrium path, a player 2 is indifferent between “abide by the rules” and “violate the rules”^2^. Accordingly, player 2 will play the two alternatives with the same probability (50%). Furthermore, player 2’s expected payoffs for accepting the bribe is *T-Y*, which is greater than 0. Therefore, player 2 will accept any bribe being offered. Given that, the expected payoffs of a player 1 who offers *T* points to his or her partner is *(1200-2T-2X)/2*, which is lower than the expected payoffs of offering nothing *(1200/2 = 600)*. That is, not bribing is the dominant strategy for player 1. In equilibrium, player 1 does not offer a bribe to player 2, and player 2 violates the allocation rule with a probability of 50%. However, a growing literature has shown that actions that violate social norms can bring the decision maker with considerable psychological costs. We anticipate that participants’ behavior will deviate from the theoretical equilibria.

### Treatments

Three treatments are conducted with the same bribery game framework. The treatments only vary in the experimental instructions. In the first treatment, the game is presented as a scholarship allocation scenario in a college^3^ (the familiar-context treatment below). In the second treatment, the game is presented as a competitive bidding scenario among firms (the unfamiliar-context treatment below). In the third treatment, the game is presented in an abstract form without any specific scenario or role (the neutral-context treatment below). Table 1 summarizes the roles and terminology for the alternatives in each of the treatments. All participants are randomly assigned to one of the three treatments. In total, 100 students participate in the familiar-context treatment, 110 students in the neutral-context treatment, and 40 students in the unfamiliar-context treatment.

**TABLE 1:** The contexts and vocabulary used in the three treatments.

**Treatments**		**Familiar context**	**Unfamiliar context**	**Neutral context**
Earnings		Scholarship	Profits	Points
Player 1’s role		Student	Bidder	Applicant
Player 1’s alternatives	Alternative 1	Make a transfer	Make a transfer	Make a transfer
	Alternative 2	No contact	No contact	No contact
Player 2’s role		Advisor	Bid-inviter	Granter
Player 2’s alternatives	Alternative 1	Abide by the rule	Abide by the rule	Abide by the rule
	Alternative 2	Violate the rule	Violate the rule	Violate the rule

Since all participants are college students, we conjecture that the college scenario is more likely to be perceived as an evocative context. Unethical decisions in this context will trigger strong emotional responses, bringing the decision maker considerable psychological costs. Consequently, corrupt conduct (offer bribe, accept bribe, or violate the rule) should be less likely to happen in the familiar-context treatment.

Another question we are curious about is whether the unfamiliar-context treatment and the neutral-context treatment may lead to different behavioral results. As discussed earlier, a meaningful but not evocative context will not trigger emotional responses. The participants in the meaningful (yet unfamiliar) context should bear the same psychological costs as in the neutral context. As a result, we conjecture that the unfamiliar-context treatment and the neutral-context treatment will produce the same behavior.

### The Attitude Surveys

In addition to the laboratory bribery game, we also conduct an independent attitude survey to measure students’ attitudes toward unethical behaviors. In the survey, we present the bribery relationship to the respondents, and then ask them to indicate their attitude on a 7-point Likert scale. Similar to the laboratory bribery game, the same interaction structure is framed with three different contexts (i.e., familiar context, unfamiliar context, neutral context). Please see the survey with familiar context below as an example^4^.

Imagine a scholarship allocation scenario in a college. In total five students applied to the same scholarship. There are 1,000 dollars available in the award pool. All the student applicants are equally qualified. **According to the college policy**, the academic advisor shall split the $1,000 dollars among the five applicants. That is to say, each of the applicants shall receive an award of $200.
However, prior to the scholarship allocation decision, one of the five students talked to the academic advisor, sent him a gift that worth $200 (secretly and privately). As return, the academic advisor announced that student as the only person who won the scholarship, distributed all $1,000 to her. All other applicants earned nothing. The interaction between the student and the academic advisor will not be discovered by others.
Please select the response that indicates the degree to which you agree or disagree with the STUDENT and the ACADEMIC ADVISOR’S activities. There is no right or wrong answer, so try hard to be completely honest in your responses. You can state your opinions accurately as the information you submit will be completely confidential.

**Table:** 

For the STUDENT:						

**1**	**2**	**3**	**4**	**5**	**6**	**7**

Extremely	Moderately	Somewhat	Not Sure	Somewhat	Moderately	Extremely
Disagree	Disagree	Disagree		Agree	Agree	Agree

For the ACADEMIC ADVISOR:						

**1**	**2**	**3**	**4**	**5**	**6**	**7**

Extremely	Moderately	Somewhat	Not Sure	Somewhat	Moderately	Extremely
Disagree	Disagree	Disagree		Agree	Agree	Agree

Besides, we also ask the respondents to indicate their sex. To the best of our knowledge, this is the first study that use independent attitude survey to complement laboratory bribery experiment.

In total 90 participants are invited to our lab to complete the survey. The 90 students are randomized into the three different contexts (with 30 respondents in each context). We adopt a between-subjects design, each respondent only participant in one of the three contexts. Given that the survey respondents and the laboratory game participants are randomly chosen from the same population, we assume that they should have similar attitudes toward unethical behaviors in the given contexts. Our design allows us to obtain measures for attitudes that are not influenced by decisions in the laboratory bribery game. Results from the attitude survey can inform us what people perceive as the “right thing to do” in each context. Ideally, the attitudes should be able to help predict people’s behavior in the bribery game experiment.

## Analysis and Results

### Attitudes Toward Corrupt Activities in Each of the Contexts

To analyze the survey data, we take people’s attitude toward unethical conduct as the dependent variable. The first independent variable is role, which has two levels: player 1 or player 2; The second independent variable is context, which has three levels: familiar context, unfamiliar context, and neutral context. We first perform a two-way 2 (role: player 1 or player 2) × 3 (context: familiar, unfamiliar, neutral) mixed measures ANOVA with repeated measures on the “role” variable (because each respondent needs to indicate their attitude toward both players). The result is presented in Table 2. From this table, we learn that both the context variable and the role variable have significant main effects on attitude. However, there is no two-way interactions between the context variable and the role variable on attitude [*F*_(2, 87)_ = 0.735, *p* = 0.482].

**TABLE 2:** Attitudes toward unethical behavior: two-way Mixed ANOVA.

**Effect**	**DFn**	**DFd**	**F-statistics**	**GES**
Context	2	87	16.483**	0.191
Role	1	87	4.561*	0.019
Context: role	2	87	0.735	0.006

*Indicates the result is statistically significant at the *p* = 0.05 level. **Indicates the result is statistically significant at the *p* = 0.01 level.

**FIGURE 3:**
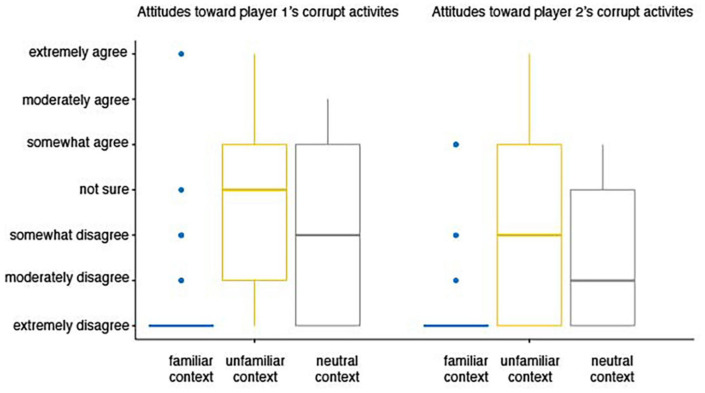
Results of the attitude surveys.

We then compare the attitudes to the two roles: The mean score toward corrupt conduct is 2.793 and 2.344 for player 1 and player 2. This difference is statistically significant (*p* < 0.001). That is, people rate player 2’s unethical behavior more negatively. We also conduct a pairwise comparison between group levels to see how context impact attitudes on each role. The result (Table 3) indicates that the mean attitude score is significantly lower in the familiar context, as compare with the other contexts. The attitude score is not significantly different in the unfamiliar-context vs. neutral context comparison. The distribution of people’s attitudes toward unethical behavior in each of the three contexts are presented in the boxplot in Figure 3. Keep in mind that because the respondents of the attitude survey did not participate in the laboratory bribery game, their responses are not influenced by the game.

**TABLE 3:** Pairwise comparison of the mean attitude toward bribing behavior.

	**Group 1**		**Group 2**				
	**Context**	**Mean (std)**	**Context**	**Mean (std)**	**Mean-difference**	***p*-value**	**Adjusted *p*-value^*a*^**
Player 1	Familiar	1.57 (1.30)	Unfamiliar	3.77 (1.83)	–2.20	0.000	0.000
	Familiar	1.57 (1.30)	Neutral	3.07 (1.78)	–1.50	0.001	0.002
	Unfamiliar	3.77 (1.83)	Neutral	3.07 (1.78)	0.70	0.105	0.316
Player 2	Familiar	1.43 (1.01)	Unfamiliar	3.00 (2.03)	–1.57	0.000	0.001
	Familiar	1.43 (1.01)	Neutral	2.60 (1.67)	–1.17	0.001	0.022
	Unfamiliar	3.00 (2.03)	Neutral	2.60 (1.67)	0.40	0.35	1

*^*a*^Bonferroni corrected *p*-value.*

### Corrupt Activities in the Laboratory Bribery Game

To analyze the data from the bribery game, we first pool all the participants’ data together, use exploratory analysis to examine how the contexts may change behavior; Next, we look at if the interaction patterns between the paired players are different across the contexts; Finally, we apply a random effect model to investigate how the contexts may impact the dynamic of decision making over time.

#### Exploratory Analysis

In general, the frequency of a player 1’s bribery attempt is 37% across all treatment, and the frequency of a player 2 violating the rule when allocating resources is 14.33%. The difference is statistically significant (Fisher exact test *p* < 0.001). That is, player 2 is less likely to engage in corrupt activities.

The frequency of a player 1’s bribery attempt is 31.13% in the familiar-context treatment, 39.81% in the unfamiliar-context treatment, and 41.83% in the neutral-context treatment. Fisher exact test results indicate that the familiar-context treatment has the lowest bribery rate (Fisher exact test *p* < 0.0001 in comparison to the unfamiliar-context and *p* < 0.0001 in comparison to the neutral-context treatment). No evidence suggests that the player 1’s bribery rate in the unfamiliar-context treatment is significantly different than in the neutral-context treatment (Fisher exact test *p* = 0.4089). Table 4 summarizes the player 1 behavior. In the familiar-context treatment, 36% of individual player 1s never tried to bribe their partners; this proportion is 20% in the unfamiliar-context treatment (significantly lower than in the familiar-context treatment; Fisher exact test *p* = 0.0480), and 17.27% in the neutral-context treatment (significantly lower than in the familiar-context treatment; Fisher exact test *p* = 0.0017). In the experiment, some of the player 1s may have selected the bribery option by mistake (or perhaps to become familiar with the game). Among all player 1s in the familiar-context treatment, 43% made bribery attempts no more than 1 time (out of 15 periods); this number is 20% in the unfamiliar-context treatment (significantly lower than in the familiar-context treatment; Fisher exact test *p* = 0.0079), and 25.45% in the neutral-context treatment (significantly lower than in the familiar-context treatment; Fisher exact test *p* = 0.0055). Moreover, the proportion of participants who constantly bribe the partners is the lowest in the familiar-context treatment (Fisher exact test *p* < 0.001).

**TABLE 4:** The frequency of the player 1s’ (potential bribers) bribing attempts.

**Never offer bribe (attempt=0/15)**			**No more than one time (attempts <= 1/15)**			**Constantly offer bribe (attempts >= 8/15)**		
**Familiar context**	**Unfamiliar context**	**Neutral context**	**Familiar context**	**Unfamiliar context**	**Neutral context**	**Familiar context**	**Unfamiliar context**	**Neutral context**
36/100	8/40	19/110	43/100	8/40	28/110	29/100	15/40	41/110
36.00%	20.00%	17.27%	43.00%	20.00%	25.45%	29.00%	37.50%	37.27%

We then compare the outcomes in the unfamiliar-context treatment and the neutral-context treatment. We do not find any significant differences (proportion of participants who never offer bribe: *p* = 0.8922; proportion of participants who offer a bribe no more than 1 time: *p* = 0.478; proportion of participants who constantly offer a bribe: *p* = 1.0).

Table 5 summarizes the frequencies of the player 2s’ corrupt activities The proportion of participants who never violate the rule is 64% in the familiar-context treatment and 17.50% in the unfamiliar-context treatment. These two proportions are significantly different (Fisher exact test *p* < 0.0001). The player 2s in the familiar-context treatment are also much more likely to abide by the rules than those in the neutral-context treatment (Fisher exact test *p* < 0.0001). The proportion of participants who never violate the rule is 17.50% in the unfamiliar-context treatment and 30% in the neutral-context treatment. Again, the difference is not statistically significant (Fisher exact test *p* = 0.147).

**TABLE 5:** The frequency of the Player 2s’ (potential bribees) unethical decisions.

**Never violate the rule (attempt = 0/15)**			**No more than one time (attempts <= 1/15)**			**Constantly violate the rule (attempts >= 8/15)**		
**Familiar context**	**Unfamiliar context**	**Neutral context**	**Familiar context**	**Unfamiliar context**	**Neutral context**	**Familiar context**	**Unfamiliar context**	**Neutral context**
64/100	7/40	33/110	74/100	9/40	56/110	5/100	4/40	12/110
64.00%	17.50%	30.00%	74.00%	22.50%	50.91%	5.00%	10.00%	10.91%

Upon completion of the bribery game, all the participants are asked to complete an open-ended questionnaire^5^ about their decisions and reasoning in the bribery game. According to the questionnaire, 54% of the participants in the familiar-context treatment indicate that corrupt behaviors are typically disapproval in the college context. Among them, only 6% engaged in corruption in the experiment.

#### Interaction Between the Paired Players

Next, we examine the interactions between the player 1 and the player 2 in a pair. We find that a reciprocal relationship between the two players is less likely to be established in the familiar-context treatment (Table 6). Specifically, 66% of the time corrupt activity never occurs (i.e., the player 1 never offers his or her partner a bribe, and the player 2 never violates the rules) in the familiar-context treatment. This percentage is 50.83% in the unfamiliar-context treatment (significantly lower than in the familiar-context treatment, Fisher exact test *p* < 0.0001) and 53.20% in the neutral-context treatment (significantly lower than in the familiar-context treatment, Fisher exact test *p* < 0.0001). Moreover, the player 2s in the familiar-context treatment are more likely to reject the bribery from the other person (Table 7). In aggregate, 81.58% of the bribes from player 1 are rejected in the familiar-context treatment. This percentage is 63.35% in the unfamiliar-context treatment (significantly lower than in the familiar-context treatment, Fisher exact test *p* < 0.0001) and 77.12% in the neutral-context treatment (significantly lower than in the familiar-context treatment, Fisher exact test *p* = 0.075).

**TABLE 6:** Proportion of pairs never commit any unethical decision.

**Corruption never happened (no bribery, no violation)**		
**Familiar context**	**Unfamiliar context**	**Neutral context**
990/1,500	305/600	863/1,650
66%	50.83%	53.20%

**TABLE 7:** Proportions of offers being rejected by player 2.

**The player 2 rejected the bribery from the player 1**		
**Familiar context**	**Unfamiliar context**	**Neutral context**
381/467	159/251	507/657
81.58%	63.35%	77.12%

In addition, we notice that some player 2s violate the game rule without an offer from the player 1. Such behavior could be understood as “signaling.” The essence of bribery relationship is a mutual exchange of favors relying on trust and reciprocity. Since the two individuals in a pair may interact with each other repeatedly, in early stage of the game, the player 2 may have an incentive to signal the player 1 that he is interested in establishing such relationship (in the hope that the player 1 will start to offer bribe in later interactions). From Table 8, we can see that only 4.16% of the interactions are initiated by player 2 in the familiar-context treatment (i.e., player 2 violates the game rules without an offer from player 1). This proportion is also the lowest among the three treatments. Again, we do not see different results in the unfamiliar-context treatment and the neutral-context treatment.

**TABLE 8:** Proportion of interactions initiated by player 2.

**The player 2 violate the rule without any bribery from player 1**		
**Familiar context**	**Unfamiliar context**	**Neutral context**
43/1033	44/349	130/993
4.16%	12.61%	13.09%

#### The Dynamic of Decision Making

To further examine whether the familiar context is inversely predicting the probability of engaging in corruption, we perform several regression analyses. In particular, consider the following random effect model:

$$y_{1it}=\alpha +\beta_{1}.familiar_{i}+\beta_{2}.unfamiliar_{i}+\beta_{3}.male_{i}+\;$$

$$U_{it}+E_{it}$$

where:

*y*_*1it*_ is player 1’s bribery decision at period t. *y*_1*it*_ = 1 if individual *i* offers a payment to the other person in period *t* and *y*_1*it*_ = 0 if otherwise.

*familiar*_*i*_ and *unfamiliar*_*i*_ are dummy variables, they indicate if individual *i* is in a particular context. We use the neutral context treatment as the compare group.

*male*_*i*_ is a dummy variable indicates if individual *i* is male.

*U*_*it*_ is the individual-specific random effect (i.e., between-entity error).

ε_*it*_ is the error term.

α is the constant term.

We first use the model above to estimate how the contexts affect player 1’s decisions, results are reported in column (1) in Table 9. Next, we add period and the group an individual is in as additional controls, and then estimate the model again. Results are listed in column (2) and (3).

**TABLE 9:** Regression analysis with random effect models.

	**(1)**	**(2)**	**(3)**	**(4)**	**(5)**	**(6)**	**(7)**	**(8)**
		**Player 1**			**Player 2**			
**Model**	**Random effect models**				**Random effect models**			
**Dependent variable**	**Offer payment**	**Offer payment**	**Offer payment**	**Offer payment**	**Rule violation**	**Rule violation**	**Rule violation**	**Rule violation**
Familiar context	–0.076***	–0.074***	–0.074***	–0.078***	–0.086***	–0.086***	–0.085***	–0.045**
	(0.016)	(0.016)	(0.016)	(0.019)	(0.012)	(0.012)	(0.012)	(0.014)
Unfamiliar context	–0.015	–0.016	–0.016	–0.030	0.031	0.031	0.031	0.064**
	(0.025)	(0.022)	(0.022)	(0.027)	(0.017)	(0.017)	(0.017)	(0.020)
Neutral context (compare group)	–	–	–	–	–	–	–	–
Amounts of points being offered	–	–	–	–	0.001***	0.001***	0.001***	0.001***
					(0.000)	(0.000)	(0.000)	(0.000)
Male	0.220*** (0.021)	0.216*** (0.021)	0.221*** (0.021)	0.204*** (0.034)	0.065*** (0.015)	0.064** (0.016)	0.067** (0.016)	0.159*** (0.025)
Familiar × male				0.015 (0.049)				–0.177*** (0.036)
Unfamiliar × male				0.060 (0.064)				–0.125* (0.047)
Constant	0.352*** (0.022)	0.306*** (0.048)	0.264*** (0.060)	0.261*** (0.060)	0.115*** (0.010)	0.066 (0.034)	0.081 (0.045)	0.058 (0.046)
Control for periods	No	Yes	Yes	Yes	No	Yes	Yes	Yes
Control for groups	No	No	Yes	Yes	No	No	Yes	Yes
Observations	250	250	250	250	250	250	250	250
R-squared	0.036	0.050	0.056	0.056	0.067	0.075	0.077	0.083

*Indicate the result is statistically significant at *p* = 0.05 level. **Indicate the result is statistically significant at *p* = 0.01 level. ***Indicate the result is statistically significant at *p* = 0.001 level.

Following that, we conduct a similar analysis for the player 2s. In addition to the existing independent variables, we add the total amount of points being offered to the model, because player 2’s decision might be influenced by how much payment was offered. Accordingly, the model becomes:

$$y_{2it}=\alpha +\beta_{1}.familiar_{i}+\beta_{2}.unfamiliar_{i}+\beta_{3}.male_{i}+\;$$

$$\beta_{4}offer_{it}+U_{it}++E_{it}$$

where:

*y*_*2it*_ is player 2’s decision at period *t*. *y*_2*it*_ = 1 if individual *i* violates the allocation rule at period t and *y*_2*it*_ = 0 if otherwise.

*offer*_*it*_ represents the amount of point being offered in period *t*.

All the other independent variables are the same as in the previous model. We also change the model specification by adding periods and groups as controls. The estimation results are shown in column (5)—column (7) in Table 9.

As Table 9 suggest, the familiar-context dummy is negatively related to the probability of engaging in corrupt activities for both players. This effect is highly robust to changes in specification. In addition, we find that male participants are more likely to engage in corrupt behavior than female participants.

Lastly, we are interested in if there is any interaction between the context effect and the gender effect. We then add the interaction terms of the gender and the contexts into the models (i.e., *familiar male*, and *unfamiliar* *male*), and then estimate the parameters. Regression results with the interaction terms are reported in column (4) and column (8) in Table 9. From the results, we do not find any interaction effect between gender and context for player 1. The interaction effect for player 2 is quite interesting. In particular, the marginal effect of being in the familiar context is –0.063 for male and is –0.045 for female. This result suggests that the familiar context makes both male and female less likely to violate the funding allocation rule, but it has a stronger effect on male than on female. The marginal effect of being in the unfamiliar context is 0.098 for male and is 0.064 for female. That is to say, compare with the neural context, the unfamiliar context makes people more likely to violate the funding allocation rule. One possible reason of this observation is that people may have higher tolerance for bribing behavior in a business competition setting. However, this finding also adds a caveat to the application of loaded context: it brings extra confounding variable into the experiment and reduce experimental control.

## Discussion

In this paper, we use bribery game as an example to look at how different contexts impact unethical decision making in laboratory economic studies. Past studies on this topic (e.g., Cooper et al., 1999; Abbink and Hennig-schmidt, 2006; Barr and Serra, 2009) often compare the distinction between neutral context and loaded context. Our work tries to extend this dichotomous view by taking into account individual’s emotional responses. In particular, we propose that emotional responses and psychological costs evoked by the framing is the key to understand when and why context may alter behavior.

We carry out three different treatments: a familiar-context treatment, an unfamiliar-context treatment, and a neutral-context treatment. In addition, we also use an independent survey to measure people’s attitudes toward unethical behaviors in each of the contexts. Since attitude is considered to be an effective indicator of behavior, observations from the survey can help us better understand the motivation of decisions. In summary, we find that the survey respondents in the familiar context express the strongest negative attitudes toward corruption. Attitudes toward unethical behavior are the same in the neutral context and the unfamiliar context. Behaviorally, corrupt activities are substantially fewer in the familiar-context treatment than in the other two treatments. In the unfamiliar-context treatment and the neutral-context treatment, we do not find essential differences in the participants’ behaviors.

From the attitude survey, our first finding is that most student respondents hold negative attitudes toward corrupt activities across all contexts (cheers for humanity!). Further, we find that although both the familiar context and the unfamiliar context are heavily loaded with suggestive words and background stories, the former clearly evoke stronger emotional responses. When we compare the attitudes in the unfamiliar context and the neutral context, we do not see statistically different outcomes. Result from the pairwise comparison analysis indicates that the negative attitudes are amplified by the familiar (i.e., college) context among the student respondents. Moreover, we find the students hold stronger negative attitudes toward player 2’s unethical behavior. Result from the mixed measures ANOVA shows that there is no interaction effect between the context variable and the role variable. Note that the survey respondents and the bribery game participants are randomly chosen from the same population, their attitudes toward bribery should be similar. Hence, we anticipant the results from the attitude survey can help predict behavior in the lab. First of all, we anticipate that the player 2s should be less likely to engage in corrupt activities than the player 1s; Secondly, we predict that the participants in the familiar-context treatment should be less likely to engage in unethical behavior; Thirdly, we expect that the unfamiliar-context treatment and the neutral-context treatment should lead to similar behavior outcome.

Findings from the laboratory bribery game confirmed all these predictions. The experimental data suggest that the possibility of engaging in unethical behavior (offer bribe, accept bribe, or violate the rule) is obviously, in a statistical sense, the lowest in the familiar-context treatment, for both the player 1s and the player 2s. The unfamiliar-context treatment and the neutral-context treatment produce the same behavior. Observations from the bribery game, together with evidence from the attitude survey, suggest that in unethical decision-making experiments, emotional responses evoked by the context can be used to explain participants’ behavior. Since the familiar context evokes the strongest emotional responses among all the contexts, the norm-consistent behaviors (i.e., behave with integrity) are more predictive in the familiar-context treatment than in the others.

With this insight, let’s try to reconcile the mixed findings from past studies. Abbink and Hennig-schmidt (2006) conduct a bribery game experiment structured as interactions between “firms” and “public officers.” Two different instructions are used, one with neutral descriptions and words and the other with suggestive words. The main finding from the study is that contexts did not change student participants’ behavior, and the authors attribute this finding to the participants’ lack of “expertise.” A similar bribery game by Barr and Serra (2009) with University of Oxford student as participants find that when the participant plays as bribee, context has no effect on bribe acceptance; meanwhile, when the participant plays as briber, the context alters the behaviors. The authors attribute these results to participants’ “intrinsic motivation.” Here, we think the aforementioned “expertise” or “intrinsic motivation” can be good explanations in their individual studies, the emotional responses evoked by the framing might provide a generic explanation for all experiments of this type. Based on our results, the experimenters can expect to observe behavior change only when the emotional responses and psychological costs evoked by dishonest practices are different across contexts.

Alatas et al. (2009) invited real public officers in Indonesian to participate in a bribery game experiment. They find that when the public officer participants play as the bribees, they are less likely to engage in unethical behavior. One interpretation is that when participants play a role that is the same as their real-life identity, they know better the consequences of their decisions. Therefore, familiarity with the experimental role would help prevent unethical behavior from happening. We consider that familiarity with the identity is a special case of familiarity with the context. Individual’s pre-game experience is not just limited to participants’ real-life identity. Rather, it is an integration of one’s real-life role, expertise, knowledge, worldview, and all factors that contribute to the individual’s self-concept. As long as a participant is familiar with the context, she will link the experimental task to her self-concept. Consequently, behaviors that violate certain social norm would trigger stronger emotional responses.

## Limitations and Further Direction

A major limitation of the current study is that the experimental design cannot fully reveal the mechanism underlying context effect in unethical decision-making experiments. For instance, there are at least two other possible explanations for the observed results. First, it is possible that the familiar context amplifies the cognitive dissonance evoked by engaging in corrupt activities. A key element that determines the intensity of the dissonance is personal involvement—the more attention one devotes to the unethical decision, the greater the dissonance experienced. As Elliot Aronson (1999) suggests: “…cognitive dissonance theory makes its strongest and clearest predictions when the self-concept of the individual is engaged. … dissonance is greatest and clearest when it involves not just any two cognitions but, rather, a cognition about the self and a piece of our behavior that violates that self-concept.” Another possible mechanism is that the familiar context changes behavior via norm salience. Cialdini et al. (1990) propose that only “activated” norms impact people’s behavior. In the current experiment, it is possible that the social norm in the familiar-context treatment is more salient to the participants. Accordingly, the participants are more likely to follow the dominant norms (i.e., behave with integrity). In future studies, it would be interesting to further investigate the how emotion, cognitive dissonance, and social norm jointly (or separately) determine behavior.

Unbalanced sample is another limitation of this paper. In particular, the sample we collected is unbalanced in two senses: First, the number of participants in the unfamiliar-context treatment is fewer than the other two treatments (40 in the unfamiliar-context treatment, 100 in the familiar-context treatment, and 110 in the unfamiliar-context treatment); Second, female participants account for 73% of the sample. To the first point, the highly unbalanced participant number is caused by administrative reasons that out of our control. Such sample may jeopardize the power of the statistic tests, especially when the variables of interest have different variances across treatments. The good news for us is that even in the unfamiliar-context treatment, the sample size (*n* = 40) is still sufficient for the statistical tests we used. Unbalanced sample may also cause unequal variances between samples. To address this concern, we compared the variances of bribing decisions in the familiar-context treatment and the unfamiliar-context treatment and find no significant difference. To the second point, the unequal number of male and female is caused by both the gender imbalance of the school and our recruitment strategy. At Jianghan University (where the experiment was conducted), female students account for 60% of the student population. Moreover, due to our sampling strategy, it turned out female students are more likely to reply to our recruitment email. Although gender effect is not the main focus of this paper, it would be better if we can use a more representative sample to conduct the study. In future studies, it would be interesting to systematically explore how gender affect the ways people interpret contexts.

Additionally, our conclusion would be much convincing with a counterfactual experiment in a non-student population. In the current study, the underlying assumption is that the familiar context (i.e., scholarship allocation) can give the student participants a more self-relevant, emotional experience than the unfamiliar context (bidding competition in business setting). This implies that with participants who is more familiar with the bidding competition in business setting but less familiar with academic setting, the contexts may lead to different behavior patterns. In the future, we hope to test our theory with different samples.

## Concluding Remarks

Social scientists from different fields (economics, psychology, sociology, political science, etc.) apply various approaches to investigate the motivation of dishonest behavior in games, yet interdisciplinary cooperation in this area is surprisingly rare. This lack of communication may result from the disagreement on some issues concerning fundamental research methodology.

Our research contributes to one of the persistent, but still far from settled questions on experimental methodology: what is the role of experimental context in laboratory unethical decision-making research? In economics, it has become standard to present the experimental tasks using the neutral-context instructions, even in the experiments that emphasize the values and ethics embedded in the context. Because people worry about the loaded-context instructions may impact behavior in an unpredictable manner. Past studies in bribery games show that the loaded context alters people’s behavior in some cases but produces the same result as the neutral context in others. Nevertheless, using loaded-context instructions has clear advantages. For instance, the participants can better learn the experimental tasks and be more engaged; the experimenters can explicitly associate the loaded contexts with the research questions to better understand participants’ motivation. By identifying factors through which the loaded context impacts behavior, we can actually use the properly framed context as a way to gain ecological validity.

We do not think our results should be seen as a whole rejection of the neutral-context design approach. Instead, the point we are trying to make is that we should always keep our experimental design as simple as possible, but not simpler. In reality, moral obligation and emotional responses play vital roles in unethical decision making; therefore, it is important to simulate these non-monetary payoffs while conducting laboratory experiments. In unethical decision-making experiments, we think it is inappropriate to assume that experimental manipulation can be studied apart from the cultural and social norms that define its meaning. When the values and ethics associated with the contexts are unclear to participants, we put the ecological validity and reproducibility of the experiment at risk.

## Data Availability Statement

The raw data supporting the conclusions of this article will be made available by the authors, without undue reservation.

## Ethics Statement

The studies involving human participants were reviewed and approved by the Jianghan University, Committee of Research Ethics. Written informed consent for participation was not required for this study in accordance with the national legislation and the institutional requirements.

## Author Contributions

SW designed and conducted the experiment and performed the statistical analysis. TC and SW together wrote the manuscript. Both authors contributed to the article and approved the submitted version.

## Conflict of Interest

The authors declare that the research was conducted in the absence of any commercial or financial relationships that could be construed as a potential conflict of interest.

## Footnotes

1 At Jianghan University, 30 RMB is approximately the cost of a one-person daily meal in the student dining hall.

2 Note that the determination of “rule-breaking” is based on the decision regarding whether or not to accept the bribe, rather than the decision regarding point allocation.

3 At the Jianghan University (and many other colleges in China), the academic advisor is in charge of scholarship allocation.

4 All the surveys are attached in the complementary materials. The original version of the surveys is in Chinese language (available upon request).

5 In the questionnaire, we ask the participants were you engaged in any bribery relationship in the game, and what is the rationale of your decision.
