# Supplementary material for: When and Why Contexts Predict Unethical Behavior: Evidence From a Laboratory Bribery Game
Source: Front Psychol. 2021 Jul 9;12:675319. doi: 10.3389/fpsyg.2021.675319 (PMC8299706; doi:10.3389/fpsyg.2021.675319)
Supplement: Supplementary file 1 [file Presentation_1.pdf]

frontiers

Supplementary Material

When and why contexts predict unethical behavior: Evidence from a laboratory Bribery Game

This file includes:

The translated version (English) of the

1. Attitude Surveys
2. Experimental instructions

Note: all the surveys and instructions here have been translated into English. The original versions (in Chinese) are available upon request.

Translated attitude survey: familiar context

Please read the following paragraphs, and then response to the questions below.

Imagine a scholarship allocation scenario in a college. In total five students applied the same scholarship. There are 1000 dollars available in the award pool. <u>All the student applicants are equally qualified</u>. **According to the college policy**, the academic advisor shall split the $1000 dollars among the five applicants. That is to say, each of the applicants shall receive an award of $200.

However, prior to the scholarship allocation decision, one of the five students talked to the academic advisor, sent him a gift that worth $200 (secretly and privately). As return, the academic advisor announced that student as the only person who won the scholarship, distributed all $1000 to her. All other applicants earned nothing. The interaction between the student and the academic advisor will not be discovered by others.

Please select the response that indicates the degree to which you agree or disagree with the STUDENT and the ACADEMIC ADVISOR'S activities. There is no right or wrong answer, so try hard to be completely honest in your responses. You can state your opinions accurately as the information you submit will be completely confidential.

For the **STUDENT**:

1<br>Extremely<br>Disagree	2<br>Moderately<br>Disagree	3<br>Somewhat<br>Disagree	4<br>Not Sure	5<br>Somewhat<br>Agree	6<br>Moderately<br>Agree	7<br>Extremely<br>Agree

For the ACADEMIC ADVISOR:

1<br>Extremely<br>Disagree	2<br>Moderately<br>Disagree	3<br>Somewhat<br>Disagree	4<br>Not Sure	5<br>Somewhat<br>Agree	6<br>Moderately<br>Agree	7<br>Extremely<br>Agree

You are: Male Female

Translated attitude survey: unfamiliar context

Please read the following paragraphs, and then response to the questions below.

Imagine a public bidding scenario in the electronic construction industry. In total five firms submitted bids for the same project. The project will generate a profit of 1000 *points* (the fictitious currency in this scenario). All the firm applicants are equally qualified. According to the industry regulation, the bid-inviter shall let the five bidders to cooperate on the project, each of them shall receive a profit of 200 *points* (1/5 of the 1000 *points*).

However, prior to the final decision, one of the five bidders talked to the bid-inviter, sent him a gift that worth 200 *points* (secretly and privately). As return, the bid-inviter announced that bidder as the only firm who won the bid. Consequently, the winning bidder earned all the 1000 *points*. All other bidders earned nothing. The interaction between the firm and the bid-inviter will not be discovered by others.

Please select the response that indicates the degree to which you agree or disagree with the Bidder and the Bid-inviter's activities. There is no right or wrong answer, so try hard to be completely honest in your responses. You can state your opinions accurately as the information you submit will be completely confidential.

For the **Bidder**:

1	2	3	4	5	6	7
Extremely Disagree	Moderately Disagree	Somewhat Disagree	Not Sure	Somewhat Agree	Moderately Agree	Extremely Agree

For the **Bid-inviter**:

1	2	3	4	5	6	7
Extremely Disagree	Moderately Disagree	Somewhat Disagree	Not Sure	Somewhat Agree	Moderately Agree	Extremely Agree

You are: Male Female

Translated attitude survey: context free

Please read the following paragraphs, and then response to the questions below.

Imagine a game where people allocate *Points* (the fictitious currency in the game). There are five *applicants* and one *granter*. 1000 *points* will be distributed among 5 applicants. All the applicants are equally qualified. According to the game rule, the granter shall split the 1000 *points* among the five applicants. That is to say, each of the applicants shall receive an award of 200 *points* (1/5 of the 1000 *points*).

However, prior to the final decision, one of the five applicants talked to the granter, sent him a gift that worth 200 *points* (secretly and privately). As return, the granter announced that applicant as the only person who won the award. Consequently, that applicant earned all the 1000 *points*. All other applicants earned nothing. The interaction between the applicant and the granter will not be discovered by others.

Please select the response that indicates the degree to which you agree or disagree with the APPLICANT and the GRANTER's activities. There is no right or wrong answer, so try hard to be completely honest in your responses. You can state your opinions accurately as the information you submit will be completely confidential.

For the **APPLICANT**:

1<br>Extremely<br>Disagree	2<br>Moderately<br>Disagree	3<br>Somewhat<br>Disagree	4<br>Not Sure	5<br>Somewhat<br>Agree	6<br>Moderately<br>Agree	7<br>Extremely<br>Agree

For the **GRANTER**:

1<br>Extremely<br>Disagree	2<br>Moderately<br>Disagree	3<br>Somewhat<br>Disagree	4<br>Not Sure	5<br>Somewhat<br>Agree	6<br>Moderately<br>Agree	7<br>Extremely<br>Agree

You are: Male Female

Translated experimental instruction (familiar context)

Experiment Instruction

Welcome to the decision-making lab of the Jianghan University of China. The purpose of this experiment is to study how people make decisions in a social interactive situation. If you pay attention and make good decisions, you may earn a considerable amount of money. Just for showing up, you have earned 5RMB. All earnings for today's tasks will be in addition to the 5RMB. You will earn "Points" through the experiment. At the concsion of the experiment, you will be paid 1RMB for every 100 points you earned. The more points you earn the more monetary payment you can get. At the end of the experiment, you will be paid your earnings privately and in cash. You will not be paid if you leave before you conclude the experiment. We guarantee that we will treat your decisions/answers with the utmost confidentiality. For the remainder of this experiment, please refrain from any communication with other participants. Please put away your cell phones.

In the first part of the experiment, we will simulate a scholarship allocation scenario. There are two roles in the experiment:

1. Student (apply for scholarship)
2. Academic advisor (allocate the scholarship)

Each participant in the experiment will be randomly assigned with one of the two roles. 10 participants will make up of a group. In each group, there are 5 students and 5 academic advisors. Each student applies for 5 different scholarships (scholarship A to scholarship E). Each academic advisor is in charge of allocating one of the 5 scholarships. In addition, each student will be randomly paired with an academic advisor. Prior to the scholarship allocation decision, the two participants in a pair can interact with each other All the interactions are anonymous. You will never know the identity of the others.

The rules for scholarship allocation:

- Each academic advisor will distribute 1000 Points among the 5 student applicants.
- Each student applicant may face three different outcomes: Full reward (1000 points), partial reward (200 points), and no reward (0 point).
- In addition, we ASSUME all the applicants are the same qualified.
- **According to the college policy**, all the applicants shall receive the same amount of award (partial award, 200 points)
- At the beginning of each round, the student will receive 200 points as initial endowment. The advisor does not have initial endowment.

Introduction to the STUDENTS:

If your role is the STUDENT, then you can imagine that you have applied five different scholarships. Meanwhile, you have the opportunity to contact with one of the academic advisors who are in charge of the scholarship allocation. The first decision you need to make, is whether or not to contact the academic advisor. For example (see the picture below): student3 have applied 5 different scholarships. In addition, she may transfer a certain amount of points (1-200) to the academic advisor who will allocate the rewards of scholarship C, in the hope that to earn the full reward (1000 points).
If you decide "do not contact the advisor", then you have no other decisions to make in this round. Please wait for the scholarship allocation outcome.
If you decide to "contact the advisor", then you will make your second decision: transfer a certain amount of points to the advisor. You will specify an integer of the range from 1 to 200 points. After your transfer decision, please wait for the scholarship allocation outcome. When all the scholarships have been allocated, you will see the feedback on how much reward you received from each of the scholarships.

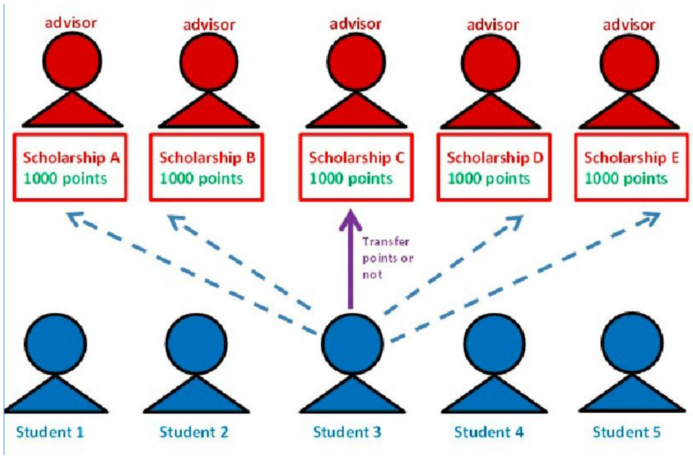

Introduction to the ACADEMIC ADVISOR:

If your role is the ADVISOR, you will allocate 1000 points among 5 student applicants. At the beginning of each round, one of the students may contact you and transfer some points to you. Based on the student's decision, you may see one of the two outcomes: (1) the student has contacted me and have transferred some points to me; or (2) the student has decided not to contact me.
If the student has contacted you, and has transferred some points to you, then you will make a selection between "I accept the transfer" and "I reject the transfer". If you accept the transfer, then the amount of offered will be deducted from the student's account and then added to your account. If you reject the offer, then both you and the student's accounts will remain unchanged. If the student has decided not to contact you, then you will make the allocation decision directly (see examples below).

Next, you will allocate the 1000 points scholarship among the 5 students. Keep in mind that all the students are equally qualified. According to the college policy, you shall split the 1000 points, and

allocate 200 points to each student. However, you can also violate the college policy, let the student in your pair earn all the 1000 points, and the other students earn nothing. In short, no matter what decision has been made by the student, you always need to make a selection between two options: (1) abide by the rule, let each student earns 200 points; or (2) violate the rule, let one student earns 1000 points, and the other students earn nothing. After that, the experiment will move to the next round.

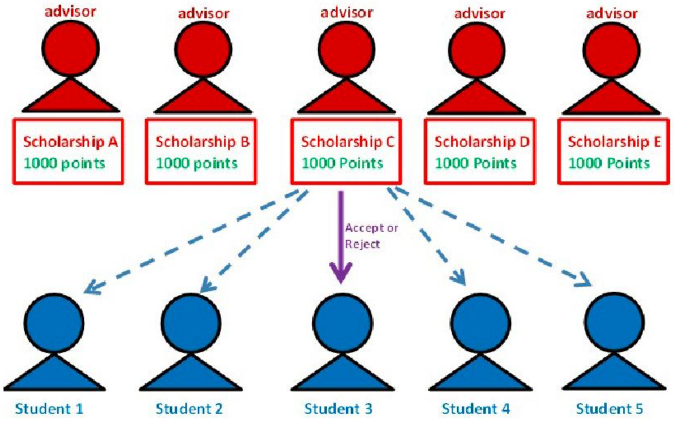

Experimental Procedure:

The game will be repeated 30 rounds. From round1 to round 15, you will play the game with a fixed partner. At the end of period 15, all the participants will be assigned to a new role, and then be paired with a new partner to play the game for another 15 periods.

How you will be paid:

At the conclusion of the experiment, four rounds will be randomly selected, two from round1-round15, and the other two from round15 to round 30. The randomly selected round will determine your earnings.

Punishment:

During the experiment, a pair of subjects will be identified as the "rule-breaking pair" if any offer from the student was accepted by the advisor. By the end of the experiment, a lottery will be played out to decide whether to punish the subjects who are in the rule-breaking pair. With a probability of 1%, the punishment occurs: both participants' earnings are cleared from their accounts.

In the second part of the experiment, you will complete a short survey independently.

Survey questions after the bribery game:

(1) When you were playing the STUDENT in the experiment, have you ever contacted the advisor?

(2) Why or why not?

(3) When you were playing the ADVISOR in the experiment, have you ever violate the rule when allocating the scholarship?

(4) Why or why not?

Translated experimental instruction (unfamiliar context)

Experiment Instruction

Welcome to the decision-making lab of the Jianghan University of China. The purpose of this experiment is to study how people make decisions in a social interactive situation. If you pay attention and make good decisions, you may earn a considerable amount of money. Just for showing up, you have earned 5RMB. All earnings for today's tasks will be in addition to the 5RMB. You will earn "Points" through the experiment. At the conclusion of the experiment, you will be paid 1RMB for every 100 points you earned. The more points you earn the more monetary payment you can get. At the end of the experiment, you will be paid your earnings privately and in cash. You will not be paid if you leave before you conclude the experiment. We guarantee that we will treat your decisions/answers with the utmost confidentiality. For the remainder of this experiment, please refrain from any communication with other participants. Please put away your cell phones.

In the first part of the experiment, we will simulate a bidding scenario in the electrical construction industry. There are two roles in the experiment:

1. Bidder (make biddings for engineering projects)
2. Bid-inviter (determine the bidding winners)

Each participant in the experiment will be randomly assigned with one of the two roles. 10 participants will make up of a group. In each group, there are 5 bidders and 5 bid-inviters. Each bidder bids for 5 different projects (project A to project E). Each bid-inviter is in charge of determining the winner of one of the 5 projects. In addition, each bidder will be randomly paired with a bid-inviter. Prior to the biding result decision, the two participants in a pair can interact with each other All the interactions are anonymous. You will never know the identity of the others.

The rules for determining the bidding result:

- Each bid-inviter will distribute 1000 Points among the 5 bidders.
- Each bidder may face three different outcomes: Single Winner (1000 points), Shared-Winning (200 points), and no Win (0 point).
- In addition, we ASSUME all the bidders are the same qualified.
- **According to the bidding policy**, all the bidders shall receive the same amount of award (shared-winning, 200 points)
- At the beginning of each round, the bidder will receive 200 points as initial endowment. The bid-inviters do not have initial endowment.

Introduction to the BIDDER:

If your role is the Bidder, then you can imagine that you have made bids for five different projects. Meanwhile, you have the opportunity to contact with one of the bid-inviter who are in charge of determining the winner. The first decision you need to make, is whether or not to contact the bid-inviter. For example (see the picture below): Bidder 3 have made bids for 5 different projects. In addition, she may transfer a certain amount of points (1-200) to the bid-inviter who will determine the winner of project C, in the hope that to be the Single winner (earn 1000 points). If you decide "do not contact the bid-inviter", then you have no other decisions to make in this round. Please wait for the bidding outcome.

If you decide to "contact the bid-inviter", then you will make your second decision: transfer a certain amount of points to the bid-inviter. You will specify an integer of the range from 1 to 200 points. After your transfer decision, please wait for the bidding results. When all the winning decision have been made, you will see the feedback on how much reward you received from each of the projects.

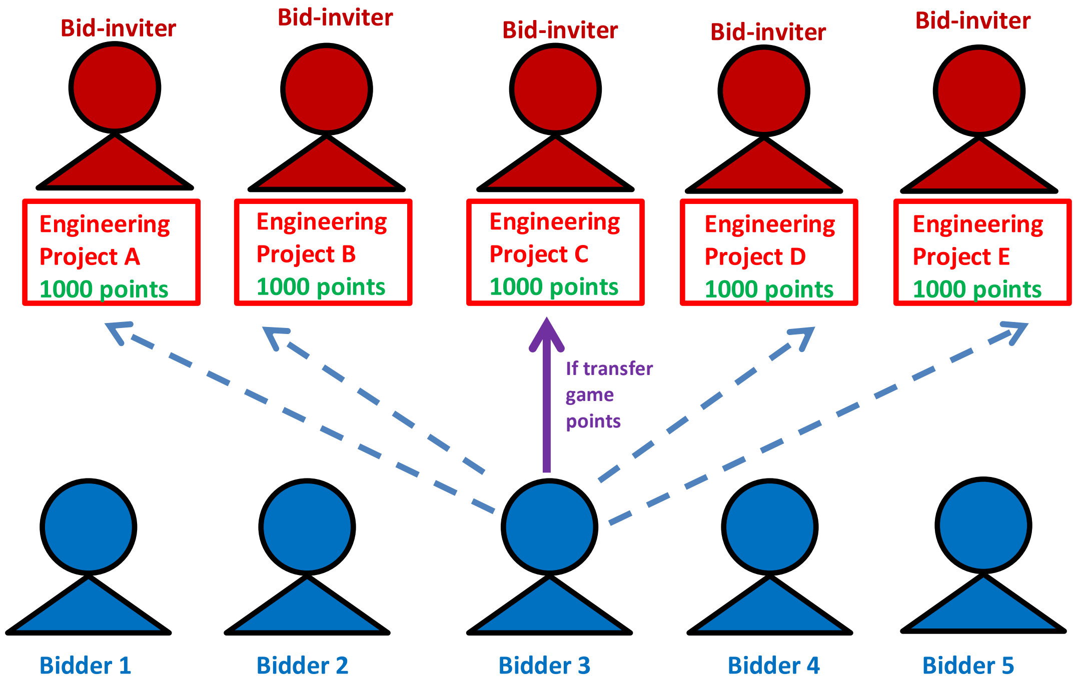

Introduction to the BID-INVITER:

If your role is the Bid-inviter, you will allocate 1000 points among 5 bidders. At the beginning of each round, one of the bidders may contact you and transfer some points to you. Based on the bidder's

decision, you may see one of the two outcomes: (1) the bidder has contacted me and have transferred some points to me; or (2) the bidder has decided not to contact me.

If the bidder has contacted you, and has transferred some points to you, then you will make a selection between “I accept the transfer” and “I reject the transfer”. If you accept the transfer, then the amount of offered will be deducted from the bidder’s account and then added to your account. If you reject the offer, then both you and the bidder’s accounts will remain unchanged. If the bidder has decided not to contact you, then you will make the allocation decision directly (see examples below).

Next, you will allocate the 1000 points among the 5 bidders. Keep in mind that all the bidders are equally qualified. According to the bidding policy, you shall split the 1000 points, and allocate 200 points to each bidder. However, you can also violate the policy, let the bidder in your pair earn all the 1000 points, and the other bidders earn nothing. In short, no matter what decision has been made by the bidder, you always need to make a selection between two options: (1) abide by the rule, let each bidder earns 200 points; or (2) violate the rule, let one bidder earns 1000 points, and the other bidders earn nothing. After that, the experiment will move to the next round.

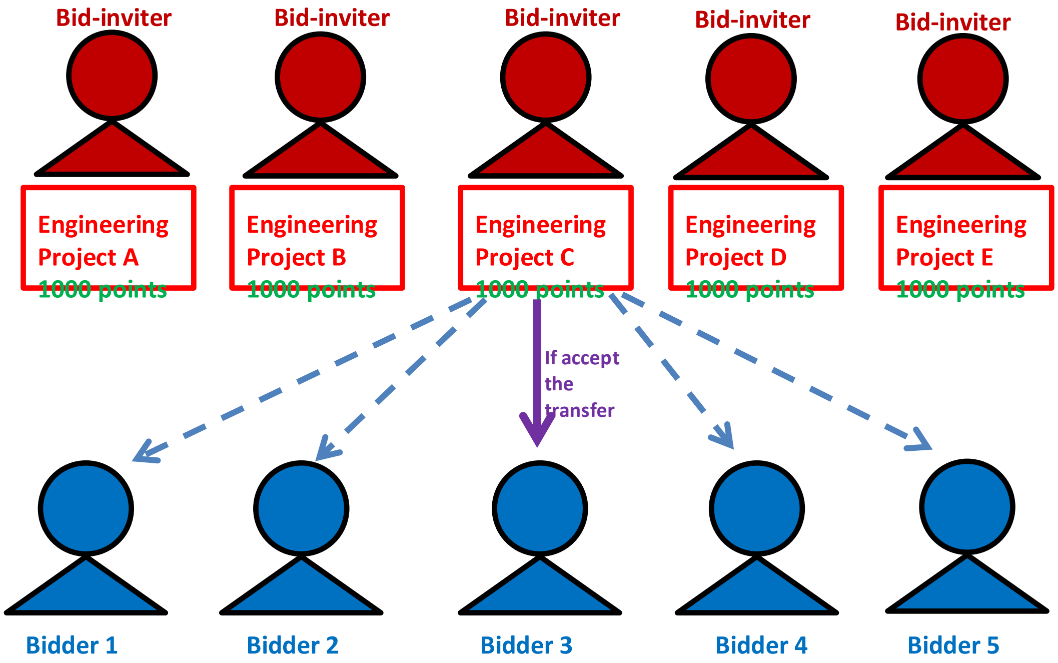

Experimental Procedure:
The game will be repeated 30 rounds. From round1 to round 15, you will play the game with a fixed partner. At the end of period 15, all the participants will be assigned to a new role, and then be paired with a new partner to play the game for another 15 periods.

How you will be paid:
At the conclusion of the experiment, four rounds will be randomly selected, two from round1-round15, and the other two from round15 to round30. The randomly selected round will determine your earnings.

Punishment:
During the experiment, a pair of subjects will be identified as the "rule-breaking pair" if any offer from the bidder was accepted by the bid-inviter. By the end of the experiment, a lottery will be played out to decide whether to punish the subjects who are in the rule-breaking pair. With a probability of 1%, the punishment occurs: both participants' earnings are cleared from their accounts.
In the second part of the experiment, you will complete a short survey independently.

Survey questions after the bribery game:
(1) When you were playing the BIDDER in the experiment, have you ever contacted the bid-inviter?
(2) Why or why not?
(3) When you were playing the bid-inviter in the experiment, have you ever violate the rule when determining the bidding outcome?

(4) Why or why not?

Translated experimental instruction (no context)

Experiment Instruction

Welcome to the decision-making lab of the Jianghan University of China. The purpose of this experiment is to study how people make decisions in a social interactive situation. If you pay attention and make good decisions, you may earn a considerable amount of money. Just for showing up, you have earned 5RMB. All earnings for today's tasks will be in addition to the 5RMB. You will earn "Points" through the experiment. At the conclusion of the experiment, you will be paid 1RMB for every 100 points you earned. The more points you earn the more monetary payment you can get. At the end of the experiment, you will be paid your earnings privately and in cash. You will not be paid if you leave before you conclude the experiment. We guarantee that we will treat your decisions/answers with the utmost confidentiality. For the remainder of this experiment, please refrain from any communication with other participants. Please put away your cell phones.

In the first part of the experiment, we will simulate a Game point allocation. There are two roles in the experiment:

1. Applicant (apply for game points)
2. Granter (allocate the game points)

Each participant in the experiment will be randomly assigned with one of the two roles. 10 participants will make up of a group. In each group, there are 5 applicants and 5 granters. Each applicant bids for 5 different projects (project A to project E). Each granter is in charge of allocate 100 game points. In addition, each applicant will be randomly paired with a granter. Prior to the biding result decision, the two participants in a pair can interact with each other All the interactions are anonymous. You will never know the identity of the others.

The rules for determining the bidding result:

- Each granter will distribute 1000 Points among the 5 applicants.
- Each applicant may face three different outcomes: Full reward (1000 points), partial reward (200 points), and no reward (0 point).
- In addition, we ASSUME all the applicants are the same qualified.
- **According to the rule**, all the applicants shall receive the same amount of award (partial rewards, 200 points)
- At the beginning of each round, the applicant will receive 200 points as initial endowment. The granters do not have initial endowment.

Introduction to the APPLICANT:

If your role is the Applicant, then you can imagine that you have made bids for five different projects. Meanwhile, you have the opportunity to contact with one of the granters. The first decision you need to make, is whether or not to contact the granter. For example (see the picture below): Applicant 3 have made bids for 5 different projects. In addition, she may transfer a certain amount of points (1-200) to the granter who will determine the winner of project C, in the hope that to receive the full reward (earn 1000 points). If you decide "do not contact the granter", then you have no other decisions to make in this round. Please wait for the allocation outcome.

If you decide to "contact the granter", then you will make your second decision: transfer a certain amount of points to the granter. You will specify an integer of the range from 1 to 200 points. After your transfer decision, please wait for the bidding results. When all the winning decision have been made, you will see the feedback on how much reward you received from each of the projects.

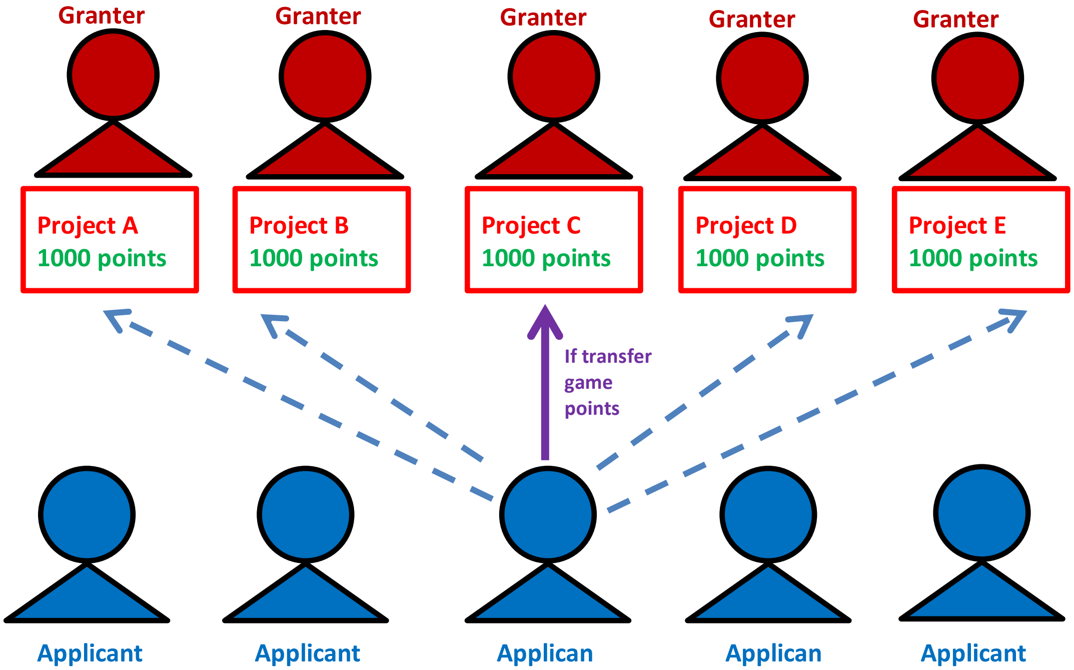

Introduction to the GRANTER:

If your role is the Granter, you will allocate 1000 points among 5 applicants. At the beginning of each round, one of the applicants may contact you and transfer some points to you. Based on the applicant's decision, you may see one of the two outcomes: (1) the applicant has contacted me and have transferred some points to me; or (2) the applicant has decided not to contact me.

If the applicant has contacted you, and has transferred some points to you, then you will make a selection between "I accept the transfer" and "I reject the transfer". If you accept the transfer, then the amount of offered will be deducted from the applicant's account and then added to your account. If you reject the offer, then both you and the applicant's accounts will remain unchanged. If the applicant has decided not to contact you, then you will make the allocation decision directly (see examples below).

Next, you will allocate the 1000 points among the 5 applicants. Keep in mind that all the applicants are equally qualified. According to the rule, you shall split the 1000 points, and allocate 200 points to each applicant. However, you can also violate the rule, let the applicant in your pair earn all the 1000 points, and the other applicants earn nothing. In short, no matter what decision has been made by the applicant, you always need to make a selection between two options: (1) abide by the rule, let each applicant earns 200 points; or (2) violate the rule, let one applicant earns 1000 points, and the other applicants earn nothing. After that, the experiment will move to the next round.

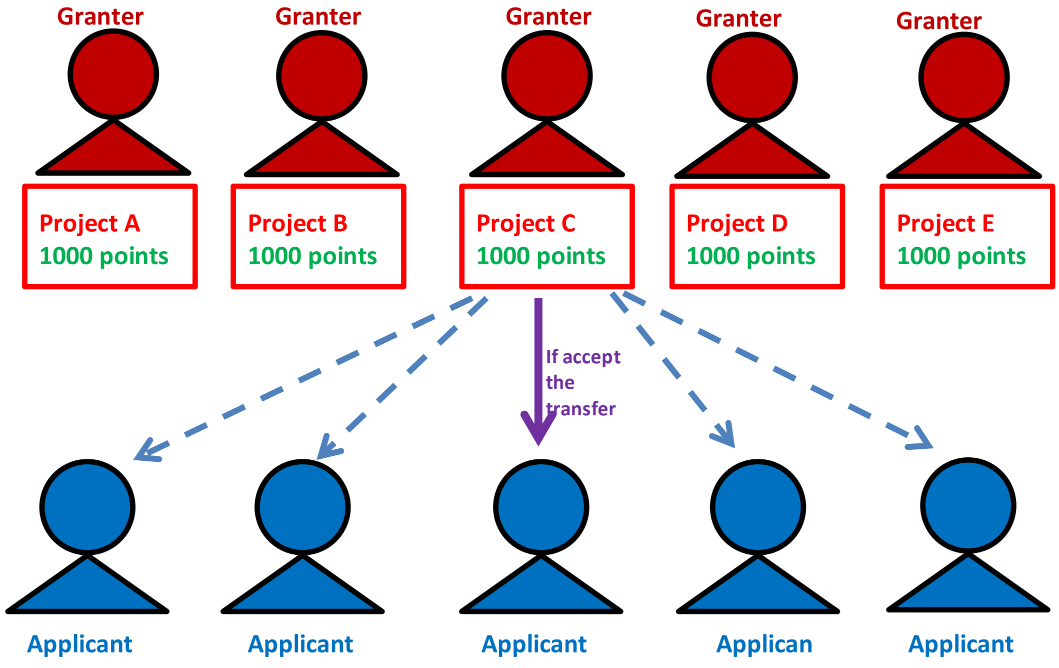

Experimental Procedure:

The game will be repeated 30 rounds. From round1 to round 15, you will play the game with a fixed partner. At the end of period 15, all the participants will be assigned to a new role, and then be paired with a new partner to play the game for another 15 periods.

How you will be paid:

At the conclusion of the experiment, four rounds will be randomly selected, two from round1-round15, and the other two from round15 to round30. The randomly selected round will determine your earnings.

Punishment:

During the experiment, a pair of subjects will be identified as the "rule-breaking pair" if any offer from the applicant was accepted by the granter. By the end of the experiment, a lottery will be played out to decide whether to punish the subjects who are in the rule-breaking pair. With a probability of 1%, the punishment occurs: both participants' earnings are cleared from their accounts.

In the second part of the experiment, you will complete a short survey independently.

Survey questions after the bribery game:

(1) When you were playing the APPLICANT in the experiment, have you ever contacted the granter?

(2) Why or why not?

(3) When you were playing the granter in the experiment, have you ever violate the rule when determining the bidding outcome?

(4) Why or why not?
